# The Characteristics of Quark Cheese Made from Buttermilk during Refrigerated Storage

**DOI:** 10.3390/foods10081783

**Published:** 2021-07-31

**Authors:** Katarzyna Szkolnicka, Izabela Dmytrów, Anna Mituniewicz-Małek

**Affiliations:** Department of Toxicology, Dairy Technology and Food Storage, Faculty of Food Science and Fisheries, West Pomeranian University of Technology, Papieża Pawła VI Str., 71-459 Szczecin, Poland; izabela.dmytrow@gmail.com (I.D.); anna.mituniewicz-malek@zut.edu.pl (A.M.-M.)

**Keywords:** buttermilk, soft cheese, texture, sensory analysis

## Abstract

The dairy industry releases huge amounts of by-products. One of them is buttermilk, obtained during butter production. This by-product is characterized by high nutritional and technological value and is finding more and more applications in food production. This study aimed to produce and analyze the characteristics of quark cheese obtained entirely from buttermilk during 3-week refrigerated (4 ± 1 °C) storage. Four kinds of sour buttermilk were used: two from industrial butter production, and another two from butter production at laboratory scale. Laboratory buttermilk differs in the kind of starter culture used in the production. The evaluation of cheese quality properties included physicochemical analyses, texture measurement, and sensory assessment. The results showed that the kind of buttermilk used in production influences the acidity, total solids, textural characteristics, and fat content of the obtained quark cheeses. All obtained cheeses had very high sensory quality throughout the storage period. The study indicates that buttermilk may be successfully used as a substitution for milk in quark cheese production.

## 1. Introduction

The dairy industry is a source of large amounts of by-products, from which one of the most abundant is buttermilk, which is currently of special interest to food technologists due to its very good technological and nutritional properties. Buttermilk is released during butter production and contains on average 3.6–6.7% lactose, 2.4–3.5% proteins (casein and serum proteins), 0.5–1.5% lipids, 0.6–0.8% ash and 0.1–0.2% polar lipids, (phospholipids and sphingolipids) originated from milk fat globule membrane (MFGM), the concentration of which is about five times higher than in whole milk [1,2,3]. This high polar lipid concentration and the presence of proteins of MFGM constituting about 19% of buttermilk proteins result in buttermilk being appreciated as a component of functional food [3,4,5]. Polar lipids have been shown to have cholesterol-lowering and anti-inflammatory effects as well as a positive impact on the nervous system functions [6,7,8]. Proteins from MFGM have antioxidant and antimicrobial properties [5,9]. Moreover, buttermilk is a good source of bioactive components such as vitamins soluble in fat, essential fatty acids, and conjugated linoleic acid (CLA) [1,3]. Buttermilk consumption also provides minerals, particularly calcium, phosphorus, and potassium, as well as vitamin B12 and riboflavin [1]. Depending on the acidity, there are distinguished two types of buttermilk, i.e., sweet buttermilk obtained during churning of unfermented cream and sour (cultured) buttermilk obtained from sour cream or by fermentation of sweet buttermilk with the cultures of mesophilic lactic acid bacteria (*Lactococcus lactis* ssp. *lactis*, *Lac. lactis* ssp. *cremoris*, *Lac. lactis* ssp. *lactis* biovar *diacetylactis*, and *Leuconostoc mesenteroides* ssp. *cremoris*). According to Eurostat [10], in 2017 more than 29% of milk processed in the European Union was used for butter production and 2.4 million tons of this product were obtained. Since the production yield is about 50% [11], buttermilk production reached a similar level. Due to the growing trend of by-product use, buttermilk is finding a wider and wider range of applications, especially in the food industry. Those applications are dictated by pro-health properties and by its low cost, emulsifying properties of MFGM components, and similarity to skimmed milk. Presently buttermilk is used for instance to improve water-binding capacity in yogurt production, or enhance crumb texture in bakery [1,12,13]. Moreover, numerous studies suggest the application of buttermilk for the production of a wide range of other products, such as fruit drinks [14,15] or fermented beverages [8,16,17]. However, one of the greatest possibilities of buttermilk application is found in cheese processing [1,3,18]. According to the literature [19,20], the incorporation of buttermilk into cheese improves its texture, viscosity, moisture retention, and sensory characteristics, as well as contributing to its nutritional properties. A positive impact of buttermilk on cheese quality is one of the reasons that it may be employed in low-fat cheese technology [18,21]. One of the approaches of buttermilk use is the production of quark cheese, a type of fresh soft cheese obtained by acid coagulation of casein. Quark cheese is characterized by low energy content and high digestibility [22]. Instead of milk, quark cheese may be produced from buttermilk with or without whey protein concentrate addition [23], from buttermilk obtained during churning of full-fat yogurt [24], or from concentrated buttermilk with cream addition [25].

Despite the favorable properties of buttermilk as a raw material for cheese production, there is a lack of literature concerning its use of as the only ingredient for quark cheese production. For that reason, we aimed to investigate whether purely buttermilk cheeses are of good physicochemical, textural, and sensory quality. Within the study, the quality of quark cheese made exclusively from buttermilk during 3-week refrigerated (4 ± 1 °C) storage was analyzed. To assess the influence of buttermilk origin on quark cheese characteristics, research included four kinds of buttermilk, two from industrial and two from laboratory-scale butter production.

## 2. Material and Methods

### 2.1. Material

Two kinds of commercial buttermilk, C1B and C2B, obtained during industrial butter production were delivered by two Polish dairy companies localized in central Poland. Those buttermilk types were obtained during the churning of sour cream and already contained strains of mesophilic lactic acid bacteria. Two other kinds of buttermilk were obtained at laboratory scale from sweet cream purchased at a local market. Collected sweet buttermilk was subsequently inoculated with two types of DVS (direct vat set) lyophilized mesophilic lactic acid bacteria starter cultures to obtain sour buttermilk. Both contained the same bacteria strains, *Lactococcus lactis* ssp. *lactis* and *Lactococcus lactis* ssp. *cremoris*, but had different manufacturers. The first culture was MO 242 produced by SACCO Srl (Cadorago, Italy) (buttermilk L1B production) and the second was MSO culture produced by Biochem Srl (Montelibretti, Italy)—buttermilk L2B. All the chemical reagents used in the study were of analytical grade.

#### 2.1.1. Buttermilk Production

Buttermilk was prepared by churning of pasteurized non-homogenized cream with 30% fat content in a laboratory-scale churning machine. Subsequently the buttermilk was pasteurized (72 °C, 10 min), cooled to ambient temperature, and divided into two batches. The first batch was inoculated with MO 242 culture and the second with MSO culture in amount of 0.6 g/L. Both batches were incubated overnight at 25 ± 1 °C and respectively L1B and L2B buttermilk were obtained.

#### 2.1.2. Buttermilk Quark Cheese Production

Cheese production consisted of the heating of buttermilk to a temperature of 40 °C. The thermal treatment caused the formation of firm cheese curd. After two hours, the curd was cut into app. 2 cm cubes, gently stirred and heated to 70 °C with the rate 1 °C/10 min. During the thermal treatment, the separation of whey from casein curd occurred. The curd was then carefully transferred into a cheese cloth with the use of a slotted spoon, allowed to drain, and pressed for 30 min in a wooden cheese press using a load of 4 kg/kg of quark. Then cheese portions, each of approximately 150 g, were vacuum packed (under the pressure 15 mbar for 2.5 s) into 40 μm PA/PE foil [26]. The obtained samples were coded respectively to the codes of buttermilk: quark cheese from buttermilk C1B–C1C, C2B–C2C, L1B–L1C and L2B–L2C, and stored at a temperature of 4 ± 1 °C for 3 weeks.

### 2.2. Methods

#### 2.2.1. Buttermilk Analyses

Buttermilk assessment included the estimation of protein, fat, and total solids content and titratable acidity [27]. Protein content was determined by Kjeldahl method and fat content by Gerber method. Total solids content was tested by drying at 105 °C until constant mass. Titratable acidity, expressed in % of lactic acid was analyzed by titration with 0.25 N NaOH solution. The pH was evaluated with a pH meter CP-411 (Elmetron, Zabrze, Poland).

#### 2.2.2. Buttermilk Quark Cheese Analyses

The cheese yield was calculated by dividing the weight of cheese directly after the production process by the weight of the buttermilk and the results were presented as kilograms of cheese per 100 kg of buttermilk [28]. After 1, 7, 14, and 21 days of refrigerated storage, the samples underwent analyses of physicochemical, textural, and sensory properties. The physicochemical analyses included the assessment of total solids content, fat content, titratable acidity in % of lactic acid [27], and pH using pH meter CP-411 (Elmetron, Zabrze, Poland). Additionally, we measured water activity a_w_ using the HygroLab device (Rotronic AG, Bassersdorf, Switzerland).

Textural properties were analyzed using texture analyzer TA.XT plus (Stable Micro System, Godalming, UK). Texture profile analysis (TPA) included the assessment of the following characteristics: hardness (the peak force during the penetration of the sample), adhesiveness (the negative peak force during the withdrawal of the probe), springiness (the distance of the detected height during the second compression divided by the original compression distance) and cohesiveness (the area of work during the second compression divided by the area of work during the first compression). The samples were penetrated with 6 mm diameter aluminum cylindrical probe to a depth of 20 mm. The test speed was 5 m/s and trigger force was 1 G.

Sensory assessment was performed by a group of 5 trained panelists in accordance with international guidelines [29,30]. The age of panelists was 25–45 years and all of them were experienced in quark cheese evaluation. Moreover, the group of evaluators underwent 2 training sessions in assessment of color, taste, odor, and texture of cheese and in detection and recognition of special tastes and odors. All the samples were encoded with a 3–digit random code and evaluators received water to clean their palates. The overall sensory quality was estimated using a scoring method and included an assessment of taste, smell, structure, and consistency as well as color of cheese. Each of the quality factors was graded from 1 point (very poor quality) to 5 points (very good quality). All the grades were summed up, and the maximal score of sensory quality was 20.

#### 2.2.3. Statistical Analyses

Physicochemical analyses were performed in triplicate and textural analyses in five repetitions. The obtained results were statistically analyzed at the significance level *p* = 0.05 using Statistica 13 Software (StatSoft Inc., Tulsa, OK, USA). Mean values and standard deviations were calculated, then the values were compared by Tukey’s HSD test.

## 3. Results and Discussion

### 3.1. Buttermilk Characteristics

**Figure 1:**
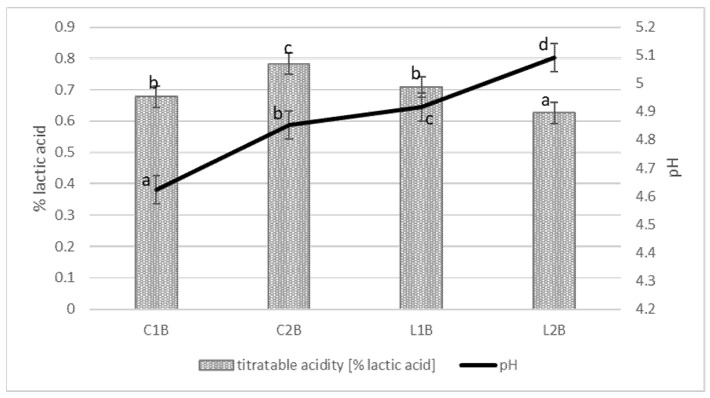
Titratable acidity and pH of tested buttermilk. C1B—commercial buttermilk 1; C2B—commercial buttermilk 2; L1B—laboratory buttermilk with MO 242 culture; L2B—laboratory buttermilk with MSO culture. Different letters indicate statistically significant differences (*p* < 0.05) between mean values.

Figure 1 presents the titratable and pH acidity of analyzed buttermilk types. Titratable acidity ranged between 0.62 and 0.72% of lactic acid. The highest titratable acidity had industrially obtained buttermilk C2B and the lowest was laboratory-obtained buttermilk with MSO culture (L2B). Buttermilk C1B and L1B had the same (*p* < 0.05) acidity. In the case of pH, all the samples had significantly different (*p* < 0.05) acidity between pH 4.62 (buttermilk C1B) and pH 5.09 (buttermilk L2B). Distinctly lower pH was noted by Gebreselassie et al. [31] in the study on naturally fermented buttermilk (pH 4.43 ± 0.18) and in a study of Ozturkoglu-Budak et al. [24] who used buttermilk with pH 4.26 for quark cheese production.

**Figure 2:**
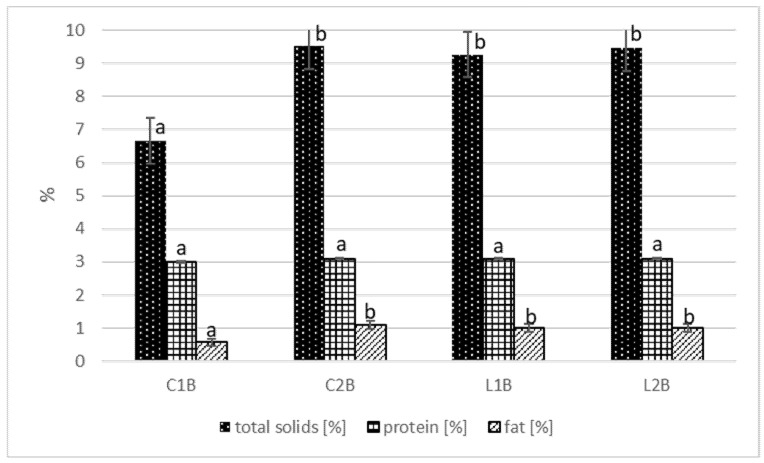
Total solids, protein and fat content of tested buttermilk. C1B—commercial buttermilk 1; C2B—commercial buttermilk 2; L1B—laboratory buttermilk with MO 242 culture; L2B—laboratory buttermilk with MSO culture. Different letters indicate statistically significant differences (*p* < 0.05) between mean values.

The composition of tested buttermilk types is illustrated in Figure 2. The buttermilk used in the study did not differ in protein level (3.0–3.1%), which is the most important component in regard of cheese production. Commercial buttermilk CB2 and both laboratory-obtained buttermilk types had the same range (*p* < 0.05) of total solids content (9.26–9.52%) and fat content (1.0–1.1%). Commercial buttermilk C1B was characterized with significantly lower (*p* < 0.05) values of these parameters (6.66% and 0.57% respectively). In the literature, a similar content of main buttermilk ingredients may be found. Gebreselassie et al. [31] stated average protein content 3.04% and fat content 1.17%. Bahrami et al. [32] stated 3.05% protein, 1.33% fat, and 10.25% total solids. The composition of the buttermilk tested by Gassi et al. [33] was 0.54% fat, 2.94% protein, and 9.16% total solids and of buttermilk tested by Bierzuńska et al. [23] was 0.4% fat and 3.3% protein. The results of all cited authors were in line with our findings. However, de Bassi et al. [17] obtained buttermilk with similar fat content (1.18%) but higher protein and total solids content (4.44% and 12.61% respectively). Distinctly higher total solids content (19.44%) was noted by Ozturkoglu-Budak et al. [24] who also used the buttermilk for quark production. The varied compositions of commercial buttermilk purchased on the market were also stated by Mituniewicz-Małek et al. [34]. Bahrami et al. [32] claimed that fat and protein are the most important components in cheese production and influence the production yield and quality of cheese. According to Gassi et al. [33] the characteristics of buttermilk depend on several factors such as the origin of milk, heat treatment of cream, and technology of butter production (e.g., slow or rapid churning). Moreover, as stated by Sodini et al. [35] the use of either sweet or sour cream for butter-churning affects buttermilk composition. The composition of analyzed buttermilk corresponds with the composition of skimmed milk, which contains about 9% of total solids and 3.3% of protein [36,37].

According to the literature, buttermilk as well as concentrated and powdered buttermilk may be successfully employed in the production of mozzarella cheese [38], pizza cheese [19], processed cheese [39], and Cheddar-style cheese [20]. In our study, we decided to analyze the possibility of using buttermilk in quark cheese production due to its short production process, important position of this kind of cheese on the market, and (important from the consumer point of view) high nutritional properties and low fat content [22,23].

### 3.2. Production Yield and Composition of Buttermilk Cheeses

The yield of quark cheese production was 27.7 ± 1.5 kg/100 kg for cheese C1C, 23.3 ± 0.9 kg/100 kg for cheese C2C, 25.4 ± 1.4 kg/100 kg for cheese L1C, and 22.1 ± 1.1 kg/100 kg for cheese L2C. Statistical analysis (*p* < 0.05) showed that production yield of C1C did not differ from L1C and production yield of C2C was in the same range as L2C. The results indicate that origin of commercial buttermilk as well as the kind of bacteria starter culture used for laboratory buttermilk fermentation have an impact on production yield. According to Farkye [40], the physicochemical properties of raw material together with the parameters of the cheese production process are crucial factors determining the yield of production and quality characteristics of obtained products. The range of production yield in our study was much higher than in the study of Bahrami et al. [32], who obtained production yield in the range 17.12–19.85 kg/100 kg. The abovementioned researchers produced cream cheese from mixes of whole milk and buttermilk, containing 0% to 50% of buttermilk. Higher production yield in our study may be the result of using 100% of buttermilk without mixing it with milk. Quoted authors concluded that the addition of buttermilk leads to higher production yield and softer and moister curd structure. Higher production yield of buttermilk cheese may be due to the ability of MFGM components, including phospholipids to retain and absorb whey in cheese curd [41]. The water-binding capacity of buttermilk in cheese production resulting in softer cheese structure was stated also by Hickey et al. [20]. Moreover, higher moisture content and production efficiency may relate to the changes of casein electric charge during butter-churning [32].

Table 1 presents physicochemical characteristics of buttermilk quark cheeses during storage. The titratable acidity was the lowest in C1C cheese from commercial buttermilk C1B (0.615–0.810% of lactic acid). Second in this parameter was L1C cheese from laboratory-obtained buttermilk with MO 242 culture. A similar range of titratable acidity had cheese C2C from commercial buttermilk C2B and cheese L2C from laboratory-obtained buttermilk with MSO culture. The percentage of lactic acid varied during the storage. Comparing the value from 1st and 21st day of storage, the titratable acidity decreased in cheese C1C, L1C and L2C. The increase was observed only in cheese C2C. The titratable acidity of buttermilk fresh cheese obtained by Bierzuńska et al. [23] was 0.7% lactic acid, which is in the same range as cheese C1C.

The pH value also varied among cheese samples and storage days (Table 1) The lowest pH on day 1 and 7 had cheese C1C and on day 14 and 21—C2C. Cheese L2C had high pH value during all storage periods. The pH of buttermilk cheese obtained by Bierzuńska et al. [23] was 4.6, which is similar to the results of C1C cheese after 1 and 7 storage days. pH of milk–buttermilk cheeses tested by Bahrami et al. [32] ranged from 4.43–4.47, which is lower than in our study. Ozturkoglu-Budak et al. [24] obtained buttermilk quark cheese characterized by much lower pH ranging from 3.91–3.96 during the 2-week storage period.

Significantly, the lowest (*p* < 0.05) total solids content was cheese C1C (Table 1), in the case of which the production yield was the highest. The highest total solids content were cheeses with lower production yield, i.e., C2C and L2C. We may conclude that high production yield relates to higher water retention in the curd, thus lower total solids content in final product. A higher level of total solids (27.01–37.52%) was obtained by Bahrami et al. [32]; however, the cheese was characterized by lower production yield. Moreover, the cited authors noticed that with the use of a higher amount of buttermilk in milk–buttermilk cheese production, total solids content is decreased, which may explain the lowest values of solids in our study. Additionally, Sakkas et al. [42] claimed that the addition of buttermilk in form of lyophilized sweet sheep buttermilk increased the moisture of reduced-fat sheep milk cheese. Similar to our study, total solid content in buttermilk fresh cheese (21.75%) was obtained by Bierzuńska et al. [23]. In turn, Ozturkoglu-Budak et al. [24] obtained buttermilk quark cheese with 21.3–25.8% of total solids. The lowest fat content (Table 1) was noted for cheese C1C, and the highest fat content were cheeses L2C and C2C. Significantly, the lowest fat content in C1C cheese is the result of significantly the lowest fat content in buttermilk C1B (Figure 2) as well as the highest production yield of this cheese and its lowest total solids content. The range of fat content in buttermilk quark cheese is distinctly lower than in whole milk quark cheese, which according to Jasińska et al. [43] ranges from 9.0–17.2%. Low fat content of buttermilk cheese relates to lower fat content in the raw material. In Bahrami et al. [32] who produced cream cheese from milk and milk–buttermilk mixes, it is presented that by substituting 50% milk with buttermilk, fat content in cheese is halved (from 10.48% to 5.18%). Much lower fat content in buttermilk cheese was noted by Bierzuńska et al. [23]; however, the cited researchers used buttermilk with lower fat content (0.4%). Ozturkoglu-Budak et al. [24] obtained buttermilk quark cheese with 3.50–3.75% fat, which is in line with our results for L1C cheese. Ferreiro et al. [25], who produced quark cheese from concentrated buttermilk and cream, obtained a product with 10.32% fat. Our study indicates that by using buttermilk as raw material we may obtain milk cheese analogues with diverse fat and total solids content. The advantage of buttermilk quark cheese is that it may contain 7 times greater concentration of phospholipids in comparison with milk quark cheese; however, phospholipids content is positively correlated with fat content in buttermilk cheese [25].

The last characteristic present in Table 1 is water activity of buttermilk cheeses. Despite differences in total solids content, no statistically significant differences (*p* < 0.05) in the water activity of the tested samples were stated. Water activity a_w_ is a parameter indicating the degree of water molecule connection with food ingredients. The value of water activity informs us about the availability of water for microorganisms and the possibility of their development, thus a_w_ strongly affects the shelf-life of the product. When the value of a_w_ is close to 1, the food product is perishable [44]. As a result of high water activity together with the presence of active lactic acid bacteria, quark cheese belongs to the group of dairy products characterized by short shelf-life [45].

### 3.3. Texture Parameters

The textural properties of buttermilk cheeses during 3 weeks refrigerated storage are presented in Table 2.

Significantly the lowest value (*p* < 0.05) of hardness was cheese C1C (Table 2), which may relate to the lowest total solids content in this product (Table 1). This cheese sample was characterized by very soft and moist structure. Significantly the highest (*p* < 0.05) hardness during all storage period were samples C2C and L2C with the highest content of total solids (Table 1). Lower hardness of cheese with higher water content was observed also by Henneberry et al. [46] and Hickey et al. [20]. The hardness of quark cheese made from milk may be varied and is related to raw material quality, technology of cheese production, and storage time [47,48]. Analyzing the literature, the hardness of quark cheese may be in the range of 2 N [49], 3 N [43], or 5 N [48]. Taking into consideration adhesiveness, the results are related to hardness values. As in the case of hardness, the lowest adhesiveness was cheese C1C and the highest C2C and L2C (Table 2). The lowest adhesiveness of C1C cheese may be due to it having the lowest fat content, because, as noticed by Mazur et al. [48], there is a positive correlation between fat content and the adhesiveness of fresh quark cheese. Springiness of all analyzed samples was similar, but C1C cheese was of lower value in this texture parameter (Table 2). The range of springiness (0.751–0.966) is close to the value for full-fat quark cheese tested by Mazur et al. [48], which was 0.88. The lowest values of cohesiveness were products C2C and L2C (Table 2). All the textural characteristics were stable during the storage time. Small variations were observed only in the case of C2C cheese (changes of hardness and adhesiveness) and L1C (springiness and cohesiveness). Observed differences in texture characteristics of tested samples suggest that the origin of the buttermilk, as well as the kind of starter culture used in cheese production, has an impact on the structure of the product.

Texture characteristics of cheese are strongly associated with consumer acceptance of the product. The studied buttermilk cheeses, due to the use of low in fat raw material, are considered to be low-fat cheeses. Poor body texture in comparison with full-fat products is one of the main disadvantages of low-fat cheese. One of the methods for improving this kind of cheese is buttermilk addition [18]. The texture improvement is a result of the high water-holding capacity of phospholipids present in buttermilk. According to Hickey et al. [20], who tested Cheddar-style cheese with buttermilk addition, a softer texture of low-fat cheese with phospholipids is associated with higher water content. The author stated that water is bounded with the *para-*casein matrix, which leads to a more porous cheese structure. Romeih et al. [21], who produced low-fat Cheddar cheese with buttermilk powder, observed a smoother and more homogenous protein curd of cheese with powdered buttermilk in comparison to control cheese with skimmed milk powder. In Borges et al. [50], concentrated by ultrafiltration, buttermilk was added to milk in amounts of 5% to produce reduced-fat ripened cheese. The obtained cheese had similar hardness and chewiness as full-fat cheese. Skeie et al. [51] stated that the addition of 15% of buttermilk and 3% of microparticulated whey proteins results in texture improvement and lower hardness value of low-fat Norwegian cheese compared to cheese without additional ingredients. However, Sakkas et al. [42] observed an increase of hardness and gumminess and a decrease of adhesiveness in reduced-fat cheese with lyophilized sweet sheep buttermilk addition. Beside cheese, buttermilk is proved to improve the texture characteristics of yogurt [13,52].

### 3.4. Sensory Assessment

**Figure 3:**
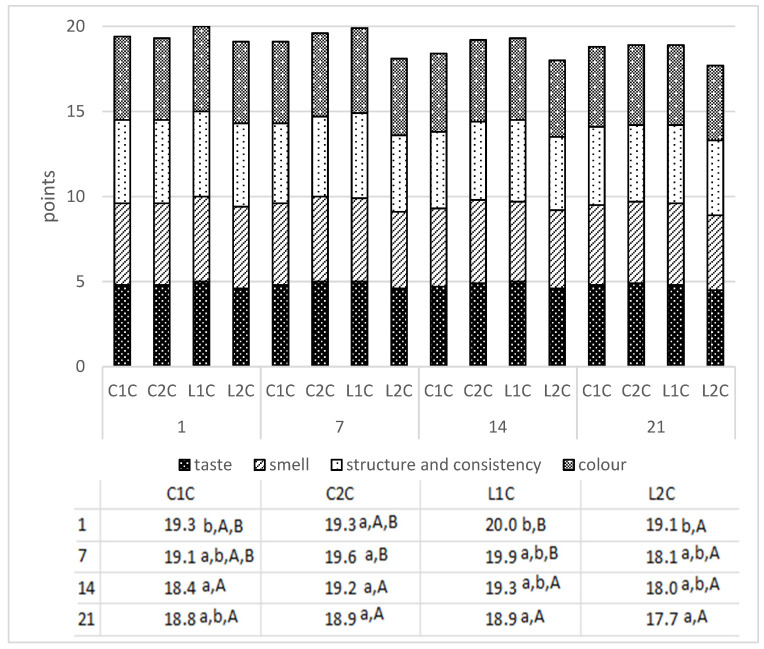
Sensory quality of buttermilk quark cheeses during the storage. C1C—cheese from commercial buttermilk C1B; C2C—cheese from commercial buttermilk C2B; L1C—cheese from laboratory buttermilk with MO 242 culture L1B; L2C—cheese from laboratory buttermilk with MSO culture L2B. Different letters in superscript indicate statistically significant (*p* < 0.05) differences between mean values in columns (lowercase letters) and in rows (uppercase letters).

The sensory properties of cheeses are key factors in their quality and help to estimate consumer’s perception and commercial success [53]. The sensory quality of buttermilk cheeses during 3 weeks refrigerated storage are demonstrated in Figure 3. The presented values are the sums of points of taste, smell, structure and consistency, and color, and maximal sensory quality score is 20 points. The results show that despite differences in physicochemical and textural parameters, all samples were characterized by high sensory quality ranging from 17.7 to 20.0 points. The tastes and smells of all cheeses were clean, aromatic, and slightly sour. The structure was fine-grained and uniform, and the consistency was smooth and soft. Color of the samples was uniform and creamy-white. The sensory quality of C2C cheese did not vary during storage time. The rest of the samples had the best sensory quality on the first day of the storage. The statistically significant (*p* < 0.05) differences among the analyzed samples were found on day 1 and 7, when cheese L2C had the lowest sensory quality. On day 14 and 21 there were not statistically significant (*p* < 0.05) differences. The results suggest that buttermilk produced in both commercial and laboratory conditions may be viewed as a good raw material for high-quality quark cheese production.

Nevertheless, the high sensory quality of the cheeses is not in line with the results of other researchers. Hickey et al. [20] tested Cheddar-style cheese made from mixes of 70% milk and 30% buttermilk. The authors stated that cheese made with buttermilk addition had worse aroma, flavor, and appearance than control milk Cheddar cheese and was characterized by a softer texture. Additionally, in the study by Bahrami et al. [32], cheeses from milk and buttermilk mixes were evaluated. The authors produced cream cheese with 0–50% buttermilk addition. They found that cheese made from more than 25% of buttermilk were not acceptable in the case of sensory characteristics due to texture and flavor decrease. Both studies pointed out the too-soft texture of cheese with buttermilk. However, in the case of quark cheese, which was the subject of our study, a soft texture is a typical sensory characteristic [54]. On the other hand, in the study by El Sayed et al. [55], the addition of 20% and 30% of buttermilk concentrate improved the organoleptic properties of processed cheese spreads. Additionally, Borges et al. [50], who tested reduced-fat ripened cheese, revealed that cheese with 5% concentrated buttermilk addition had similar sensory characteristics as full-fat cheese during a 90-day ripening period. Good sensory properties of buttermilk quark cheese processed at 100°C, comparable with properties of quark cheese made of skimmed milk, were noted by Ozturkoglu-Budak et al. [24]. The cited authors described buttermilk quark cheese as creamy and mildly acidic, and chose it as the most preferable sample.

## 4. Conclusions

Buttermilk, one of the most abundant by-products of the dairy industry, is produced in growing amounts in the European Union. A large body of the literature has proved its excellent nutritional and health-promoting properties, as well as high potential in food industry applications. This study aimed to determine the possibility of using buttermilk as a substitute for milk in quark cheese production. Quark cheese is a kind of fresh cheese made through acid coagulation of milk performed by mesophilic lactic acid bacteria. This product, due to low calorie content and wide range of culinary applications, is very popular in many European countries. The study showed that buttermilk may be effectively used to produce high-quality quark cheese with appropriate properties as well as a very good sensory quality throughout 3 weeks refrigerated storage. The source of buttermilk and a kind of starter culture influenced acidity, total solids and fat content, and the texture of obtained products, especially hardness and adhesiveness. The developed buttermilk cheese may be viewed as a product with high commercial potential and may be successfully implemented by dairy industry.

## Figures and Tables

**Table 1:** Physicochemical properties of buttermilk quark cheeses during the storage.

Days of Storage	Product			
	C1C	C2C	L1C	L2C
titrable acidity [% of lactic acid]				
1	0.795 ± 0.026 ^c,A^	1.005 ± 0.026 ^a,B^	1.080 ± 0.045 ^a,B^	1.185 ± 0.026 ^b,C^
7	0.810 ± 0.000 ^c,A^	1.005 ± 0.052 ^a,C^	0.915 ± 0.026 ^b,B^	1.140 ± 0.026 ^b,D^
14	0.525 ± 0.026 ^a,A^	1.185 ± 0.026 ^b,C^	0.915 ±0.026 ^b,B^	0.960 ± 0.026 ^a,B^
21	0.615 ± 0.026 ^b,A^	1.125 ± 0.045 ^b,D^	0.885 ± 0.026 ^b,B^	1.020 ± 0.026 ^a,C^
pH				
1	4.60 ± 0.01 ^a,A^	4.78 ± 0.01 ^b,B^	4.86 ± 0.01 ^a,C^	5.13 ± 0.01 ^c,D^
7	4.63 ± 0.01 ^a,A^	4.87 ± 0.01 ^c,B^	4.85 ± 0.01 ^a,B^	5.07 ± 0.01 ^b,C^
14	5.09 ± 0.06 ^c,D^	4.72 ± 0.01 ^a,A^	4.84 ± 0.01 ^a,B^	4.98 ± 0.01 ^a,C^
21	4.93 ± 0.01 ^b,C^	4.76 ± 0.01 ^b,A^	4.85 ± 0.01 ^a,B^	5.07 ± 0.00 ^b,D^
total solids [%]				
1	11.59 ± 0.28 ^a,b,D^	21.71 ± 1.26 ^b,B^	18.4 ± 0.30 ^a,C^	25.19 ± 0.75 ^a,A^
7	12.15 ± 0.21 ^a,C^	27.46 ± 0.34 ^a,A^	17.55 ± 2.04 ^a,B^	26.29 ± 0.32 ^a,A^
14	11.41 ± 0.31 ^b,C^	25.71 ± 0.33 ^a,A^	17.66 ± 1.29 ^a,B^	27.09 ± 1.28 ^a,A^
21	11.14 ± 0.08 ^b,D^	20.96 ± 0.12 ^b,B^	18.45 ± 0.76 ^a,C^	25.62 ± 0.42 ^a,A^
fat content [%]				
1	1.0 ± 0.0 ^a,A^	5.5 ± 0.5 ^a,C^	3.5 ± 0.0 ^a,B^	7.0 ± 0.0 ^a,D^
7	1.0 ± 0.0 ^a,A^	7.3 ± 0.3 ^c,C^	3.2 ± 0.3 ^a,B^	7.0 ± 0.0 ^a,C^
14	1.0 ± 0.0 ^a,A^	6.5 ± 0.0 ^b,C^	3.5 ± 0.0 ^a,B^	7.2 ± 0.3 ^a,D^
21	1.0 ± 0.0 ^a,A^	5.5 ± 0.0 ^a,C^	3.3 ± 0.3 ^a,B^	6.8 ± 0.3 ^a,D^
water activity (a_w_)				
1	0.961 ± 0.016 ^a,A^	0.967 ± 0.015 ^a,A^	0.970 ± 0.016 ^a,A^	0.968 ± 0.017 ^a,A^
7	0.967 ± 0.015 ^a,A^	0.960 ± 0.017 ^a,A^	0.967 ± 0.016 ^a,A^	0.963 ± 0.016 ^a,A^
14	0.970 ± 0.019 ^a,A^	0.960 ± 0.012 ^a,A^	0.972 ± 0.014 ^a,A^	0.974 ± 0.010 ^a,A^
21	0.969 ± 0.018 ^a,A^	0.954 ± 0.015 ^a,A^	0.965 ± 0.016 ^a,A^	0.966 ± 0.016 ^a,A^

C1C—cheese from commercial buttermilk C1B; C2C—cheese from commercial buttermilk C2B; L1C—cheese from laboratory buttermilk with MO 242 culture L1B; L2C—cheese from laboratory buttermilk with MSO culture L2B. Different letters in superscript indicate statistically significant (*p* < 0.05) differences between mean values in columns (lowercase letters) and in rows (uppercase letters).

**Table 2:** Texture parameters of buttermilk quark cheeses during the storage.

Days of Storage	Product			
	C1C	C2C	L1C	L2C
hardness [N]				
1	0.133 ± 0.015 ^a,A^	1.660 ± 0.245 ^a,C^	1.024 ± 0.052 ^a,B^	1.910 ± 0.133 ^a,C^
7	0.168 ± 0.007 ^a,A^	3.459 ± 0.091 ^b,D^	1.244 ± 0.327 ^a,B^	1.974 ± 0.129 ^a,C^
14	0.175 ± 0.015 ^a,A^	3.600 ± 0.308 ^b,D^	0.956 ± 0.137 ^a,B^	2.402 ± 0.298 ^a,C^
21	0.193 ± 0.041 ^a,A^	1.871 ± 0.097 ^a,C^	0.946 ± 0.094 ^a,B^	1.925 ± 0.309 ^a,C^
adhesiveness [g.s]				
1	−14.370 ± 4.010 ^a,A^	−124.530 ± 50.203 ^a,A^	−109.215 ± 7.406 ^a,A^	−225.620 ± 83.248 ^a,B^
7	−19.875 ± 1.069 ^a,A^	−362.434 ± 47.678 ^b,D^	−93.086 ± 22.455 ^a,B^	−170.473 ± 35.494 ^a,C^
14	−19.939 ± 5.096 ^a,A^	−303.685 ± 68.519 ^b,C^	−91.309 ± 9.940 ^a,A,B^	−185.289 ± 72.622 ^a,B^
21	−23.654 ± 3.741 ^a,A^	−152.031 ± 11.601 ^a,B,C^	−116.131 ± 17.767 ^a,B^	−185.619 ± 38.231 ^a,C^
springiness				
1	0.751 ± 0.133 ^a,A^	0.911 ± 0.046 ^a,B^	0.928 ± 0.013 ^b,B^	0.966 ± 0.034 ^a,B^
7	0.881 ± 0.038 ^a,A^	0.952 ± 0.031 ^a,A^	0.901 ± 0.006 ^a,b,A^	0.922 ± 0.058 ^a,A^
14	0.813 ± 0.054 ^a,A^	0.941 ± 0.044 ^a,B^	0.886 ± 0.032 ^a,A,B^	0.944 ± 0.049 ^a,B^
21	0.807 ± 0.058 ^a,A^	0.938 ± 0.007 ^a,B^	0.935 ± 0.020 ^b,B^	0.899 ± 0.038 ^a,B^
cohesiveness				
1	0.619 ± 0.055 ^a,B^	0.346 ± 0.090 ^a,A^	0.574 ± 0.026 ^b,B^	0.491 ± 0.125 ^a,A,B^
7	0.610 ± 0.029 ^a,A^	0.408 ± 0.109 ^a,A^	0.926 ± 0.045 ^c,B^	0.409 ± 0.172 ^a,A^
14	0.587 ± 0.040 ^a,B^	0.345 ± 0.078 ^a,A^	0.447 ± 0.033 ^a,A,B^	0.326 ± 0.131 ^a,A^
21	0.515 ± 0.035 ^a,B^	0.399 ± 0.020 ^a,A^	0.497 ± 0.036 ^a,B^	0.360 ± 0.085 ^a,A^

C1C—cheese from commercial buttermilk C1B; C2C—cheese from commercial buttermilk C2B; L1C—cheese from laboratory buttermilk with MO 242 culture L1B; L2C—cheese from laboratory buttermilk with MSO culture L2B. Different letters in superscript indicate statistically significant (*p* < 0.05) differences between mean values in columns (lowercase letters) and in rows (uppercase letters).

## Data Availability

Not applicable.
